# Adverse mental health outcomes in breast cancer survivors compared to women who did not have cancer: systematic review protocol

**DOI:** 10.1186/s13643-017-0551-2

**Published:** 2017-08-14

**Authors:** Helena Carreira, Rachael Williams, Martin Müller, Rhea Harewood, Krishnan Bhaskaran

**Affiliations:** 1 Department of Non-Communicable Disease Epidemiology, Faculty of Epidemiology and Population Health, London School of Hygiene & Tropical Medicine, Keppel Street, London, WC1E 7HT UK; 2 Clinical Practice Research Datalink (CPRD), Medicines and Healthcare products Regulatory Agency, London, UK; 3 Department of Emergency Medicine, Inselspital, Bern University Hospital, University of Bern, Bern, Switzerland

**Keywords:** Breast neoplasms, Survivors, Mental health, Mental disorders, Systematic review, Protocol

## Abstract

**Background:**

Recent increasing trends in breast cancer incidence and survival have resulted in unprecedented numbers of cancer survivors in the general population. A cancer diagnosis may have a profound psychological impact, and breast cancer treatments often cause long-term physical sequelae, potentially affecting women’s mental health. The aim of this systematic review is to identify and summarise all studies that have compared mental health outcomes in breast cancer survivors, versus women who did not have cancer.

**Methods:**

This study will be a systematic review of the literature. Four databases, including MEDLINE and PsycINFO, will be searched to identify potentially relevant studies. The search expressions will use a Boolean logic, including terms for the target population (women who have had breast cancer), outcomes (psychiatric disorders) and comparators (e.g. risk, hazard). All mental disorders will be eligible, except those with onset normally occurring during childhood or strong genetic basis (e.g. Huntington disease). The eligibility of the studies will be assessed in two phases: (1) considering the information provided in the title and abstract; (2) evaluating the full text. Studies including women diagnosed with breast cancer 1 year or more ago and that provide original data on mental health outcomes will be eligible. Studies in which all women were undergoing surgery, chemotherapy or radiotherapy, or hospitalised or institutionalised, will be excluded, as well as studies that include patients selected on the basis of symptomatology. Two investigators will do the screening of the references and the data extraction independently, with results compared and discrepancies resolved by involving a third investigator when necessary. Study quality and risk of bias will be assessed across six broad domains. Results will be summarised by outcome, and summary measures of frequency and/or association will be computed if possible.

**Discussion:**

This review will summarise the evidence on the mental health outcomes of women who have been diagnosed with breast cancer. This information can be used to motivate further research and increase understanding of the most common mental health conditions affecting this growing population of women.

**Systematic review registration:**

PROSPERO CRD42017056946

**Electronic supplementary material:**

The online version of this article (doi:10.1186/s13643-017-0551-2) contains supplementary material, which is available to authorized users.

## Background

Survival from breast cancer increased markedly during the last decades [1]. In 2005–2009, 5-year age-standardised net survival was higher than 85% in North America and between 71 and 87% in 29 European countries [1]. Considering that breast cancer is the most frequent malignancy diagnosed in women worldwide, after non-melanoma skin cancer [2], this has already translated into an unprecedentedly large number of breast cancer survivors in the general population. Many women find the diagnosis a traumatic experience [3], and the usual reactions include anxiety, hopelessness, anger and negative and suicidal thoughts [4, 5]. Some of the treatments can also cause severe long-term suffering. For example, surgery usually results in a lifelong scar and may cause breast shape alteration, persistent pain and/or lymphoedema [6–8]. The diagnosis and treatment of the breast cancer might also affect the woman’s family, including intimacy with their partners [9] and relationships with their offspring [10]. Women who return to work may also face new challenges, not only in the relationship with their work colleagues [11] but also in their cognitive functioning [12, 13]. Women must also deal with the fear of cancer recurrence and death [14]. All of these factors may have a long-term negative impact on the mental health of breast cancer survivors.

Several systematic reviews summarised the frequency of selected mental health outcomes in oncological patients under and post-treatment [15–22]. Two reviews focused on breast cancer survivors [16, 22]. Howard-Anderson et al. [22] focused on younger breast cancer survivors (<50 years at diagnosis), an important group but who represent a small proportion of all breast cancer survivors. The systematic review by Maass et al. [16] reported prevalences of anxiety between 18 and 33% and of depression between 9 and 66%; however, most of the studies included in this review did not involve a comparison group, and therefore, it is unclear how the figures compare to those of women who did not have cancer. The range of adverse mental health outcomes in breast cancer survivors is also unlikely to be limited to anxiety and depressive disorders alone. Other outcomes, such as sleep disturbances, have been reported as frequent during the treatment period and afterwards [23, 24], and very little is known about the long-term impact of these in breast cancer patients.

The overall aim of this study is to identify and summarise studies that have quantitatively compared mental health outcomes in breast cancer survivors of at least 1 year since diagnosis, versus women who did not have cancer. Specifically, through summarising such studies, this systematic review will:

- Identify mental disorders that may be associated with a history of breast cancer
- Summarise and, where possible, synthesise quantitative estimates of associations between breast cancer history and a range of specific psychiatric outcomes
- Summarise the instruments used to evaluate mental disorders or their severity in breast cancer survivors

## Methods

This systematic review protocol follows the guidance outlined by the Preferred Reporting Items for Systematic review and Meta-Analysis Protocols (PRISMA-P) [25]. Additional file 1 provides information for each item of the PRISMA-P checklist. This review has been registered in the International prospective register of systematic reviews (PROSPERO 2017:CRD42017056946).

### Eligibility

#### Inclusion criteria

Manuscripts reporting studies satisfying the following criteria will be eligible for inclusion:

- Based on original data.
- Uses any observational study design (i.e. cohort, case-control, cross-sectional designs).
- Includes adult women (≥18 years) diagnosed with breast cancer and who survived the first year after the diagnosis.
- Includes a population-based adult female comparison group with no prior cancer.
- Provides data on at least one of our pre-specified mental health outcomes of interest, namely the following: anxiety disorders; bipolar and related disorders; disruptive, impulse control and conduct disorders; feeding and eating disorders; mood disorders; neurocognitive disorders; neurotic disorders; personality disorders; schizophrenia spectrum and other psychotic disorders; sexual dysfunctions of a psychological nature; sleep-wake disorders; somatoform disorders; substance-related disorders (including alcoholism); and trauma- and stressor-related disorders. Studies providing data on self-injurious behaviour (including self-harm, suicide and suicidal ideation) will also be included. These outcomes were selected by reviewing the list of mental disorders available in the *Diagnostic and Statistical Manual of Mental Disorders, 5th edition* [26] and the *ICD-10 Classification of Mental and Behavioural Disorders* [27].

#### Exclusions

Articles will be excluded according to the following criteria:

- Review articles, editorials, commentaries, conference abstracts, case reports and studies involving animals.
- Studies in which the selection of the breast cancer survivors depended on symptoms (e.g. *only* patients with persistent pain or fatigued) or on a mental health outcome (e.g. *only* women with depression).
- Studies which *only* presented data for the first year after the breast cancer diagnosis; however, studies following women from diagnosis may still be eligible if outcomes at ≥ 1 year or more since diagnosis are reported separately.
- Studies in which all breast cancer patients remain under treatment for cancer (except for long-term endocrine therapy) at the time of outcome ascertainment.
- Studies in which all women are institutionalised (e.g. hospitalised or in hospices).

### Search strategy

We will consider as potentially eligible all studies published in the journals indexed in MEDLINE, PsycINFO, the Cumulative Index to Nursing and Allied Health Literature (CINAHL) and the Social Sciences Citation Index, since the inception of each database up to when the database was last updated at the time of the search. A search expression will be defined with a Boolean logic, including terms for the target population (breast cancer patients), outcome (psychiatric disorder) and comparators (risk, hazard, etc.). The search expression used in MEDLINE includes terms for Medical Subject Headings (MeSH) as well as key text words with truncation to allow for variations in terminology (Table 1). The search expression will be adapted to each database, to take into account the specificities of the search algorithms.

**Table 1:** MEDLINE search expression, via OVID®

1	exp Breast Neoplasms/
2	(breast and (cancer* or carcinoma* or tumo?r* or neoplas*)).mp. [mp=title, abstract, original title, name of substance word, subject heading word, keyword heading word, protocol supplementary concept word, rare disease supplementary concept word, unique identifier]
3	1 or 2
4	exp catatonia/ or exp depression/ or exp self-injurious behavior/ or exp anxiety/
5	mental disorders/ or exp anxiety disorders/ or exp “bipolar and related disorders”/ or exp “disruptive, impulse control, and conduct disorders”/ or exp dissociative disorders/ or “feeding and eating disorders”/ or anorexia nervosa/ or binge-eating disorder/ or bulimia nervosa/ or pica/ or exp mood disorders/ or exp motor disorders/ or neurocognitive disorders/ or amnesia/ or cognition disorders/ or auditory perceptual disorders/ or mild cognitive impairment/ or consciousness disorders/ or delirium/ or dementia/ or exp neurotic disorders/ or exp personality disorders/ or exp “schizophrenia spectrum and other psychotic disorders”/ or sexual dysfunctions, psychological/ or exp sleep wake disorders/ or exp somatoform disorders/ or exp substance-related disorders/ or exp “trauma and stressor related disorders”/
6	(depressi* or dysthymia or catatonia or self-injur* or self-injury or self-injurious or self-mutilation or “self mutilation” or suicid* or self-harm or “self harm” or “self injury” or anxious* or anxiety or (panic adj1 (disorder# or attack#)) or catastrophi* or (mental adj1 (disorder or disorders)) or phobia or phobic or neurotic or (compulsive adj1 disorder) or bipolar or neurotic or (personality adj1 disorder) or psychotic or psychosis or paranoid or delusional or (sexual adj1 (disorder or dysfunction or problem#)) or insomnias or (sleep adj1 (disorder or dysfunction or problem#)) or somatoform or (substance adj3 (disorder or problem#)) or stress ajd3 disorder or (adjustment adj3 disorder)).mp. [mp=title, abstract, original title, name of substance word, subject heading word, keyword heading word, protocol supplementary concept word, rare disease supplementary concept word, unique identifier]
7	4 or 5 or 6
8	(prevalence# or frequenc* or incidence# or risk or rate* or ratio or odds or epidemiolog* or percent* or outcomes or hazard).mp. [mp=title, abstract, original title, name of substance word, subject heading word, keyword heading word, protocol supplementary concept word, rare disease supplementary concept word, unique identifier]
9	3 and 7 and 8
10	Humans/
11	Animals/
12	10 and 11
13	11 not 12
14	9 not 13

We will restrict the search to studies including humans. We will not apply any time, geographic or language restriction. If a study is published in a language not sufficiently understood by the authors, we will seek assistance to translate/understand the content.

Backwards and forward citation tracking will also be used to identify additional potential eligible studies that were not captured by the database searches.

### Data management and selection process

All records will be imported into EndNote X7 (EndNote X7, Thomson Reuters, NY, USA), and studies identified as duplicates by the software will be removed. A backup of the search expression and the records obtained from each database, as well as the date of last update and run, will be saved.

The references will be screened in two consecutive phases by two authors (HC and MM, or HC and RH). In the first phase, the title and the abstract of each study will be read to determine their eligibility for the study by applying the pre-defined inclusion and exclusion criteria (see the “Eligibility” section above). If the information provided in the title and abstract does not allow the unequivocal exclusion of the study, the full text will be considered. In the second phase, the full text of each study considered eligible in the first phase will be obtained and read in order to determine the eligibility considering all the information in the paper. The studies will be reassessed for data extraction.

The decisions taken independently by each of the investigators will be compared, and discrepancies will be resolved, involving a third investigator when necessary (RW or KB). The agreement between the two investigators will be calculated (kappa statistics).

If more than one study reports data on the same study population, we will include only the study providing data for the largest sample; if the sample size is the same, we will consider the study providing more detailed information on outcomes (e.g. results stratified for age or type of treatment received) and consider both studies for abstraction of information on the participants’ characteristics (e.g. age, menopausal status, stage at diagnosis).

A record of excluded/included studies, with the respective exclusion criterion, will be kept, and the selection process including numbers excluded at each stage for different criteria will be summarised in a flow chart.

### Data extraction

Two authors (HC and MM, or HC and RH) will extract data from each included study into a pre-defined form in Microsoft Office Excel (2013). The form will be piloted using four studies and adapted if necessary. Information will be collected on (1) study characteristics (e.g. authors, year of publication, country where the sample was obtained or duration of follow-up if applicable); (2) characteristics of the breast cancer survivors (details on participant recruitment, sample size, demographics, distribution of stage at diagnosis, time since diagnosis and type of treatments); (3) characteristics of the women who did not have cancer (recruitment of the participants, sample size, demographics); (4) information on the mental health outcomes (name of the mental condition, diagnostic criteria, instruments applied); and (5) quantitative information on the mental health outcome (e.g. prevalence or mean/median score in each group and/or relative risk comparing groups) and variables considered as potential confounders.

If a prospective study provides data for more than one point in time, we will abstract all available information.

The data extracted by each author will be compared and discrepancies resolved by consensus or involving a third researcher (KB or RW) if necessary.

### Risk of bias in individual studies

We will evaluate study quality and risk of bias in the original studies by assessing the main domains identified by Sanderson et al. as important for observational study quality and bias assessment [28], informed by the “STrengthening the Reporting of OBservational studies in Epidemiology (STROBE)” guidelines [29]. These domains are: methods for selecting study participants, methods for measuring the exposure and the outcome variables, design-specific sources of bias (excluding confounding), methods to control for confounding, statistical methods (excluding confounding) and conflict of interest [28]. Within each of the above domains, individual studies will be rated as at high risk of bias, low risk of bias or unclear risk of bias, following the Cochrane Collaboration approach formulated for clinical trials [30].

### Data analysis and synthesis

The results will be reported according to the PRISMA guidelines [31]. Tables and descriptive text will be used to summarise study characteristics and results, stratified by outcome and likely sources of heterogeneity (e.g. study design, type of population).

Quantitative synthesis of results (meta-analysis) will only be attempted for selected outcomes where deemed appropriate, taking into account the number of studies available, study designs and methods and equivalence of outcome measures and effect estimates used. Where quantitative synthesis is attempted, the DerSimonian and Laird method [32] will be used to compute summary estimates of the association between breast cancer and the discrete psychiatric outcome in question, along with 95% confidence intervals. Sub-group analyses by time since diagnosis will be conducted if possible. Prospective studies providing data for two or more time points after the first anniversary of diagnosis will be included once in meta-analysis; the relative risk estimate for the first eligible time point will be chosen. Heterogeneity will be quantified using Higgins and Thompson’s *I*-squared statistic [33]. The meta-analysis will be repeated excluding any studies identified as at high risk of bias in the quality assessment. For outcomes deemed suitable for meta-analysis as described above, funnel plots and Egger’s regression asymmetry test [34] will be used to assess publication bias and small study effects if more than ten studies are available [35].

## Discussion

The number of women who have had breast cancer is higher than ever before. These women may face many challenges when trying to assimilate back into life following their cancer diagnosis and treatment, and it is imperative to understand the long-term psychological consequences. This systematic review aims to provide a comprehensive overview of the associations between breast cancer history and mental health conditions.

Most reviews on the topic have been restricted to studying the prevalence of depression among cancer patients [15, 20]. We opted for considering a much broader list of mental disorders that have their onset during adulthood as outcomes, to give a more comprehensive picture of the spectrum of mental disorders that may affect breast cancer survivors. We also chose to include only studies in which a comparison group was available, so that the relative frequency or severity of these conditions compared to the general population could be studied.

We will include studies in which women were diagnosed with breast cancer at least one year prior to outcome measurement. Women who completed breast cancer treatments with curative intent (i.e. surgery, chemotherapy and/or radiotherapy) are often considered as survivors; however, the precise point in time when the treatments end is frequently unknown and a widely accepted definition of cancer survivor does not exist [36]. Researchers commonly use a fixed point in time to capture, in a pragmatic way, the moment at which the main course of treatment is likely to have been completed. At 1 year after the diagnosis, the vast majority of women are expected to have completed the main treatments and many have returned to their pre-cancer routines. The effect of having been diagnosed and treated for breast cancer may also vary over time [37], and thus, an adequate characterisation of the risk of mental disorders requires a known time since diagnosis.

Studies involving mental health outcomes are prone to selection bias. We will report the characteristics of the samples involved in the original studies, including the details on the recruitment of the participants. We will also evaluate and report the risk of bias and use this information to help interpret the results.

Mental disorders largely interfere with the functioning of the patients and are leading causes of disability worldwide [38]. The mean prevalence of depression among women who had breast cancer has been described in the range between 10 and 20%, depending on the methods used to evaluate it [15]. This indicates that the burden of at least depressive disorders in this population is far from negligible. The impairments caused by depression are likely to be higher in these women than in women with depression alone [39].

Even though there are several pharmacological and non-pharmacological treatments available, mental disorders are often undiagnosed and untreated. The results of this review can be used to inform health professionals about the range, frequency and severity of mental disorders among breast cancer survivors.

## Additional file

Additional file 1: PRISMA-P 2015 checklist. Checklist of the compliance of the systematic review protocol with the guidance established by the “Preferred Reporting Items for Systematic review and Meta-Analysis Protocols (PRISMA-P)” statement. (DOCX 38 kb)

## Abbreviations

MeSH: Medical Subject Headings
PRISMA: Preferred Reporting Items for Systematic Reviews and Meta-Analyses
PRISMA-P: Preferred Reporting Items for Systematic review and Meta-Analysis Protocols
PROSPERO: International prospective register of systematic reviews
